# Editors’ Book Table

**Published:** 1874-12

**Authors:** 


### Editors' Book Table.

[NOTE.—All works reviewed in the columns of the CHICAGO MEDICAL JOURNAL may be found in the extensive stock of W. B. KEEN, COOKE & CO., whose catalogue of Medical Books will be sent to any address upon request.]

*The Chicago Medical Register and Directory for* 1874–5, published under the direction and supervision of the Medico-Historical Society, has just made its appearance, and we are sorry to say, fully demonstrates the unfortunate results of the errors of organization committed by the Society, and indicated specifically in the JOURNAL, in June last. We are utterly unable to perceive in what respect this Directory differs from—so far as its special contents are concerned—or is in any manner superior to the City Directory. It is furnished, indeed, at a smaller cost, but the amount of information contained in it is smaller, and the price seems to be apportioned to the bulk rather than to the character of the contents.

The publication of this book, under such auspices, must prove a source of deep regret to all who seriously desire the elevation of the standard of their profession. The Chicago Medico-Historical Society, as a body, has done its best, the best of which a body so organized was capable; from it nothing better could be expected; from the large number of medical gentlemen of intelligence and high standing, who constitute an integral portion of the society, much more was to be expected; they were expected to display an amount of moral force to resist outside pressure far greater than could be exercised by individuals in editing a work of this character.

Unfortunately for the profession, the work itself is a standing testimonial to their misguided charity (?) and bad judgment. They have sacrificed their profession to the selfish aims of individuals, and have assumed responsibilities whose weight will press heavily upon them hereafter. The initial division of the work is entitled "Physicians in Chicago who are in good and regular standing, according to the Code of Ethics of the American Medical Association," which title is further elaborated by an explanatory "Resolution adopted by the Chicago Medico-Historical Society, August 2nd, 1874;" and is followed by an alphabetical list of, quasi, exponents of the text, remarkable, first, for its admissions; secondly, for its omissions; thirdly, for its gross injustice in admitting some and omitting others belonging strictly to the same class; and lastly, for the utter incongruity between text and exposition.

To sustain this criticism, there is ample evidence; it would not even be necessary to go outside of the membership of the Society for witnesses to prove its truth and justice.

But passing by the unjust discrimination which has been exercised toward those hitherto considered of doubtful, or even decidedly bad, repute, we will venture to suggest that Hospitals and their care and management, and the indication of their programmes of medical service, are quite as much within the comprehension of a Medical Directory as are Life Insurance Companies; and that the publication of a physician's hours of attendance upon the latter—for which he is compensated—and the refusal to publish those, upon the former—which are gratuitous—is a distinction more nice than wise, in view of possible suspicions which the maliciously disposed might, unjustly, entertain.

The book is the index of a retrograde movement in professional ethics, to use no harsher but perhaps more appropriate language—a triumph of radicalism over conservatism; from the consequences of which, recovery will be difficult and tedious.

We have no intention nor desire to impugn the motives of the gentlemen to whom we have referred, as constituting a majority of the Society; but must frankly say that their course has been marked by weakness and bad judgment from the beginning to the end, the results of which can only be demoralizing in their tendency.

It is of course unnecessary to commend the work of the Editor and Publishers, which would commend itself sufficiently, were it not amply assured, in advance, by the names of the gentlemen in charge of those duties respectively. H.

*Infant Diet.* By A. JACOBI, M.D., Clinical Professor of Diseases of Children, College of Physicians and Surgeons, New York. Revised, enlarged, and adapted to popular use by MARY PUTNAM JACOBI, M.D. New York: G. P. Putnam's Sons. 1874.

The author, or authors, of this little volume, while admitting at the outset that the milk of the mother is the best food for the child, appear to make the admission under a mental protest, in view of the many examples of women who have assumed the duties and responsibilities of maternity, although unfitted therefor, by reason of imperfect, or diseased, physical organization.

A large portion of the work consists of an excellent resume of the physiology of digestion, and the chemico vital relations of infant food; followed by descriptions of some of the pathological conditions resulting from the imperfect performance of this function. This portion of the work is unobjectionable. To some of the conclusions to which the authors have arrived, in their general summary, we must beg leave to differ very respectfully with even so high an authority as Dr. Jacobi.

We can perceive no authority in physiology which sanctions, but abundant authority in practical experience which condemns, the administration of farinaceous food to infants—food which these authors regard as essentially valuable; nor can we consider the addition of brandy and whisky to the dietary list of such young subjects, otherwise than as a practice fraught with probable, almost certain, danger to the delicate nerve structure of the infant. In this relation, we refer, of couse, as do the authors, to children in a normal condition; in morbid conditions, the indications must be appropriately met. But that an infant "in very hot weather," even "in the hottest days only," should have a teaspoonful of whisky daily with its food and water, is a plan which might be safely conducted under such experienced observation as that of the author, but which, in the majority of cases, could only result disastrously. H.

*Electro-Therapeutics: A Condensed Manual of Medical Electricity.* By D. F. LINCOLN, M.D., Physician to the Department of Diseases of the Nervous System, Boston Dispensary. Philadelphia: Henry C. Lea. 1874.

Until electricians shall have reduced the phenomena with which they are concerned under some general laws, which shall be based upon fixed principles, and shall have applied thereto a system of nomenclature which shall possess the merit of philological harmony, the study of the science of electro-therapeutics will continue to be a "vanity and vexation of spirit" to all save expert physicists. Just so long as such terms as "force" and "fluid," "force" and "cause," "current" and "intensity," are used synonymously, the science, to whose gateways these terms are the pass words, must ever remain a terra incognita to the medical student.

Dr. Lincoln's little book is one of the best manuals upon the subject which has appeared, and to those engaged in the study of electro-therapeutics, it will be found useful and instructive. It is a praiseworthy effort to reduce order out of chaos, and we have no fault to find with the effort, but must still regret the chaotic condition of the science itself, which defies the efforts of ordinary readers to comprehend it. H.

*Clinical Lectures on Various Important Diseases ;* being a collection of the Clinical Lectures delivered in the Medical Wards of the Mercy Hospital, Chicago. By NATHAN S. DAVIS, A.M., M.D., Professor of Principles and Practice of Medicine, and of Clinical Medicine, in Chicago Medical College. Edited by FRANK H. DAVIS, M.D. Second Edition. Philadelphia: Henry C. Lea. 1874.

The first edition of this little work was so fully noticed in the JOURNAL, that it is only necessary for us to refer to this as exhibiting evidences of revision, and marks of improvement over its predecessor, which appears to have met the wants of a large class of readers, if the rapid demand for a second edition may be assumed as an indication of their appreciation of its value, and of the author's popularity, while its contents and style are exponents of his skill, method and culture, as physician, teacher and scholar.

H.

*Ligation of Arteries.* By Dr. L. H. FARABŒUF, Aide D'Anatomie a la Faculte, Ancien Interne des Hopitaux de Paris. Translated by JOHN D. JACKSON, M.D., of Danville, Kentucky. With engravings. Philadelphia: J. B. Lippincott & Co. 1874.

"An operative manual," and an excellent specimen of that most excellent class of books at this time coming so prominently into notice, and supplying to the general practitioner what was omitted to a great extent in his curriculum of undergraduate studies, and what he fails to find in necessary detail in his standard text-books. The book is divided into two parts, of which the first, comprising three chapters, treats of principles, or, what the author terms generalities, *i.e.*, the "discovery of the sheath of an artery," the isolation of the vessel, and its ligation. This little book is by no means a treatise on the "art of surgery made easy" for ignorant pretenders, for the author insists at the outset, upon a competent knowledge of anatomy, and addresses his subsequent instructions to those who possess that prerequisite. Amongst the preliminary steps to the study of the ligation of an artery, the author advises that its track be carefully traced upon the skin, and suggests the best material for this purpose, *i. e.*, "tincture of iodine." After this follow most minute practical directions for holding the bistoury for making the incision, and for the discovery of the sheath, all of which occupy an entire article. The method of accomplishing the isolation of the vessels both with the bistoury and director, are amply detailed in the second article, while the third is devoted to the subject of ligation proper, both of arteries in their continuity and of those cut transversely. A chapter is also devoted to the means of obliterating arteries besides the ligature, the author's preference being decidedly for the latter.

The second, and by far the larger, portion of the book is devoted to the subject of special ligations of the vessels of the upper and lower aortic system. The anatomical and surgical directions are minute and clear. The illustrations are excellent and original.

Taking the book as a whole, it will be difficult to find a more complete and useful manual upon any subject, and in its peculiar department we know of none comparable with it. H.

*Surgical Emergencies, Together with the Emergencies Attendant on Parturition, and the Treatment of Poisoning.* A Manual for the use of General Practitioners. By WILLIAM PAUL SWAIN, F.R.C.S., Surgeon to the Royal Albert Hospital, Devonport. With eighty-two illustrations. Philadelphia: Lindsay & Blakiston. 1874.

One of the most useful little books which has issued from the medical press during the current year. "It is intended to," and does, "supply a wide-spread want," "viz., a small book containing directions for the immediate treatment of all those various emergencies with which the general practitioner may be called to deal at any moment." The contents of the book are comprised in twelve chapters, and are illustrated freely and fully. The style in which the book is written, deserves the highest commendation, as one less concise would have been inadequate to the comprehension of such a "multum in parvo." We will venture to suggest an addition to the treatment of epistaxis contained in the first chapter, *i. e.*, compression of the facial arteries upon the jaw, the efficacy of which in arresting this hæmorrhage has been demonstrated so frequently. H.

*Physicians' Visiting Lists for* 1875.

The pioneers in the publication of these little books, Messrs. Lindsay & Blakiston, are, as usual, first in the field with their issue. They can truthfully claim the propriety of the criticism, "a book which no physician can afford to do without," one which every physician reads, yes, studies, daily, even if he reads no other, and which to many is "the most interesting work ever issued from the medical press." H.

*The Nature of Gunshot Wounds of the Abdomen, and their Treatment: based on a review of the case of the late James Fisk, Jr., in its medico-legal aspects.* By EUGENE PEUGNET, M.D., Surgeon to the North-Western Dispensary; Member of the New York Pathological Society, etc., etc. New York: William Wood & Co. 1874.

The object of the publication of this monograph, as set forth in the author's preface, was to demonstrate "the necessity of a change in the manner of conducting criminal investigations and in the introduction of expert testimony." The case of Fisk, of which the history is comprised in the first chapter, is made the text for the argument which comprehends four points: 1. "The description of shock;" 2. "Penetrating gunshot wounds of the abdomen;" 3. "The physiological and toxical action of morphine;" 4. "The medical jurisprudence of the Stokes case."

Dr. Peugnet in his little monograph proves himself an expert in medical jurisprudence, no less than in physiology. He appears to have detected, with remarkable clearness, the weak points in the management of the case, both of the prosecution and defense, and his physiological analysis of the symptoms of the patient, under the varying conditions, which characterized the brief period intervening between the assault and his death, is most elaborate, learned and convincing. Beginning the examination of Dr. Peugnet's work with a decided prejudice against his conclusion, based upon inferences drawn from the published testimony in the case of Fisk, believing fully that the theory of morphine poisoning was merely an ingenious ruse devised by the counsel for the defense to divert the attention of the jury from the direct evidence of murder in the case, we have finished its examination with our prejudices removed by the light of additional evidence, and the author's brilliant physiological arguments.

While, however, the arguments of the author are convincing in support of the theory of opium poisoning—and it cannot be reasonably doubted that the immediate cause of the death of Fisk was the morphine administered after the reception of his wounds—it must still be also maintained that it cannot be reasonably doubted that he would have died from the wounds received, had no morphine been given, and that the defendant was guilty morally, if not technically, of murder in the first degree; this point, however, is beyond our province to discuss. H.

*The Philosophy of Spiritualism and the Pathology and Treatment of Mediomania.* Two Lectures by FREDERIC R. MARVIN, M.D., Professor of Psychological Medicine and Medical Jurisprudence in the New York Free Medical College for Women. Read before the New York Liberal Club, March 20, 27, 1874. New York: Asa H. Butts & Co., publishers. 1874.

The author of these lectures at the outset states his propositions most clearly, and boldly throws down the gauntlet of defiance to the defenders of spiritualism—whose philosophy he assumes to analyze—in these words: "I reject all forms of materialism, and among them spiritualism." Commencing with a denunciation of spiritualism as the "materialism of materialism," he proceeds to the argument of his lecture, and endeavors to show "that the soul cannot be material substance." The first step in his argument being the demolition of "the rhetorical nebulosities" of spiritualism as expounded by Andrew Jackson Davis and Judge Edmonds, *i. e.:*

1. That man is composed of three substances, body, spirit, and soul.

2. That the soul is the body of the spirit, and the visible body the body of the soul.

3. That the soul is a result of physical organization.

4. That within the physical man there is a spiritual man, corresponding in form and size with its fleshy exterior.

5. That spirit possesses the properties of matter.

Of which our author says, very justly, "if this is not materialism there is no such thing as materialism."

Of the spiritualistic manifestations, so-called, the author protests against their relegation to the domain of the supernatural, and insists that observers await patiently the development of scientific principles which may underlie them, believing that the delay will not be long, "for it is already clear that much, if not most of the 'spiritual phenomena' are the results of deliberate imposture, pitiable credulity or grievous disease."

Dr. Marvin has a logical mind, and exhibits evidences of study and culture in his special department; it is to be regretted that the scope of his studies has been restricted in other directions, and that in consequence he should have given utterance to language for which ignorance is no palliation, *i. e.*, "in mediæval times they (men) worshiped crucifixes, pictures of the Madonna, and strings of beads." Where is the evidence of this to be found except in the false assertion of persons as ignorant as himself on this subject? And further, the assertions that the "lives of saints, priests (whom he classes with ecstatics and media) are so many records of sexual derangement," that "St. Theresa and St. Catherine of Sienna were nymphomaniacs;" are utterances unworthy of a Christian gentleman, which, by courtesy, we assume the author to be, and grossly insulting to the intelligence of two hundred millions of human beings whose faith, that of those whom he so foully maligns, is to-day the only substantial bulwark against the encroachments of the very delusions which he so vigorously assails, and of whom some few, at least, are his peers in intellectual capacity, in logical acumen, and in purity of life.

It is to be regretted that the author's proclivity to dogmatize should so frequently manifest itself, and essentially impair the usefulness of a book, which, had its spirit been more comprehensive and its tone broader, would have served to throw much light upon the subject matter of which it treats. While all must admit the very great influence of derangements of the sexual function in the development of insanity, few, we think, will endorse the author's dogma—"Tilt the organ (the uterus) a little forward—introvert it, and immediately the patient forsakes her home, embraces some strange and ultra ism—Mormonism, Mesmerism, Fourierism, Socialism, oftener Spiritualism;" and further, "Allow the disorder to advance, and it becomes a chronic malady, and alas! the once intelligent, cultivated and pure woman sinks, through a series of strange isms and remarkable affinities, until she reaches the despicable level of the *demi-monde.*"

To the initial propositions of the book, notwithstanding their dogmatism, we most heartily subscribe, and regret that they have not been sustained with the force and perspicacity of which they were susceptible, inasmuch as they were advanced and maintained with the good intent to rescue the human mind from the terrible delusion spiritualism, with which intent we most heartily sympathize. But we cannot endorse the almost exclusive ascription of this deplorable mental condition, rightly termed insanity by the author, to sexual derangement.

The book is on the whole well worth reading, containing much that is interesting; its faults being the necessary results of hasty generalization from insufficient data. H.

*Manual of Eye Surgery.* By A. J. Howe, A.M., M.D., Author of a Treatise on Fractures and Dislocations, and Professor of Surgery in the Eclectic Medical Institute, Cincinnati. Printed by Wilstach, Baldwin & Co. 1874.

The careful reader, after opening this book, will be struck with the peculiar reasons advanced in the beginning of the preface, by the author, for its compilation: "I found the ground well covered with elaborate treatises," and later, "the general practitioner was left without a work adapted particularly to his wants." On the next page the same general practitioner "is presumed to have a lexicon to which he can refer for the definition of rare and complicated terms," and he is further assured that "the learner obtains more information from a definition found in the dictionary than at first sight might be supposed," and exhorted not to be "discouraged with the array of 'hard' words."

After such a preface the reader, at all doubtful of his philological attainments, commences an inspection of the book with some trepidation, and closer study of its therapeusis suggests the reference of a certain expert, to a "bushel of spoiled eyes." One of the chief merits of the book, as of wit, is its brevity—and the reader is tempted to add to the "Finis" the old couplet: H.

"Since so early I was done for,
I wonder what I was begun for."

*Erysipelas and Child-Bed Fever.* By THOMAS C. MINOR, M.D. Cincinnati: Robert Clarke & Co. 1874.

Multum in parvo. In this little volume Dr. Minor has attempted to show the intimate relations between the two diseases which constitute the title to his little volume, and although we cannot speak upon this subject from an unprejudiced standpoint, having long since arrived at the same conclusions, we feel sure that an attentive study of the author's data and reasoning will force any candid mind to adopt his conclusions.

His studies of the correspondence in the climatic relations of the two diseases are replete with interest, and his conclusions deduced from these we have been able personally to verify during a residence of some years in the extreme South, where the immunity from the two diseases was most striking when compared with the fatality resulting from both in this locality.

The precautions recommended as necessary to those engaged largely in obstetrical practice, in order to avoid all risk of contagion, are wise and prudent, and thoroughly justified by the mortality observed where these precautions have been neglected.

H.

*Address delivered before the American Academy of Dental Science, at their Third Annual Meeting, held in Boston, September* 20, 1874. By Dr. W. W. ALLPORT, of Chicago. Published by vote of the Academy.

Dr. Allport is too well known, by the majority of readers of the JOURNAL, to require introduction. The present address is a thoughtful *resume* of the relations between the merely mechanical and scientific or higher professional branches of what is popularly denominated Dentistry. In it is very clearly shown that between the simple making of artificial teeth, plates, etc., and the diagnosis, prophylaxis and treatment of dental diseases, there is as broad a distinction as that existing between the surgeon and the maker of splints and surgical cutlery; or as that between the physician and the nurse, electrician, bather, cupper and bleeder of old time.

In his opinion, Mechanical Dentistry should be remitted to the artisans, whilst Dental Surgery and Practice should be recognized as really a specialty of medical and surgical practice.

Perhaps this may appear, at first blush, as a matter of little import, but on reflection it will be seen to involve important practical results.

Prominently this: Dental colleges, as separate organizations, are unnecessary. The scientific dentist requires all the elementary teaching given, ordinarily, in the medical colleges, just as does the oculist, the dermatologist, or any other specialist. Dental pathology and practice may be taught by an additional chair in any medical college, remitting, as before stated, manipulations to the dental mechanic.

Our space forbids any considerable extracts from this valuable *brochure*, but to bring the practical suggestion fully before the colleges and the profession, we append the closing paragraphs of the address, with our full endorsement: A.

Let dental mechanics be otherwise taught, as a high mechanical art, and the calling fixed in the mind of the public as such, and, in a few years, a patient would as soon go to the maker of artificial legs for advice or treatment, in conservative surgery, or regarding amputation, as to the *dentician*, or *dentificier*, for advice or services in the saving of his natural teeth, or their extraction.

To drop the teaching of mechanical dentistry in private offices and in our colleges, would, in a few years, permanently divide the practice, and very soon each town of any considerable size, would have one or more of these practitioners, who, relying entirely upon success in this calling for support, and becoming personally responsible for what they did, would seek to redeem and elevate this particular art to the highest degree attainable, thereby enhancing the respectability and usefulness of their calling. And the *dentologist* would, by the broad and comprehensive teachings of medicine, become more thoroughly grounded in its science, and be better qualified to take his rank with the other medically recognized specialists. With this thorough groundwork laid, he would not only be better prepared to treat from a medical standpoint the diseases belonging to his province, but also to grapple successfully, by general treatment, with those hidden and hitherto illy understood influences which serve to prevent perfect dental development, and also to counteract those pathological conditions which act as causes of disease in the teeth and tend to break down their tissues.

With the development of this higher mission of our profession, there will be no occasion for the spectacle of dentology, with the grimace and shuffle of the mendicant, approaching the gates of the medical profession, and with downcast eyes, begging a crumb of recognition. But with the accomplished separation of the two callings, heretofore combined in our practice, dentology, enriched by the experience and the special literature of the last half century, and the foundation of its practice laid exclusively in the science of medicine, rather than divided between that and a trade, the incongruity of the past will, in a few years, disappear, and by deriving its nourishment from the body of which it is a branch, it will become more and still more assimilated to the science and the practice of medicine, and without demand, there will, both by the public and the medical faculty, be accorded, not to individual practitioners, but to the branch, a full and cordial recognition as a specialty in medicine, which will attract more generally to its ranks, as to an agreeable and useful field of labor, men of earnestness, ability and culture, the peers of any in an honorable profession.

BOOKS RECEIVED.

*Report of the Chicago Relief and Aid Society, of Disbursements of Contributions for the Sufferers of the Chicago Fire.* Printed for the Chicago Relief and Aid Society at the Riverside Press. 1874.

*Therapeutics and Materia Medica; A Systematic Treatise on the Action and Uses of Medicinal Agents, including their Description and History.* By ALFRED STILLE, Professor of the Theory and Practice of Medicine, and of Clinical Medicine, in the University of Pennsylvania, etc., etc. Fourth edition. Thoroughly revised and enlarged, in two volumes. Vol. I. Philadelphia: Henry C. Lea. 1874.

*Materia Medica, for the Use of Students.* By JNO. B. BIDDLE, Professor of Materia Medica and General Therapeutics in the Jefferson Medical College, etc., etc. Sixth edition. Revised and enlarged, with illustrations. Philadelphia: Lindsay & Blakiston. 1874.

*The Nature and Treatment of Venereal Diseases, with Numerous Cases, Formulæ and Clinical Observations.* By ROBERT A. GUNN, M.D. New York: Asa K. Butts & Co., Publishers. 1874.

*Croup in its Relations to Tracheotomy.* By J. SOLES COHEN, M.D., Lecturer on Laryngoscopy and Diseases of the Throat and Chest, in Jefferson Medical College. Philadelphia: Lindsay & Blakiston. 1874.

*The Complete Hand-book of Obstetric Surgery; or, Short Rules of Practice in Every Emergency, from the simplest to the most formidable Operations connected with the Science of Obstetrics, with numerous illustrations.* By CHAS. CLAY, M.D., late Senior Surgeon and Lecturer on Midwifery, St. Mary's Hospital, Manchester, etc., etc. From the Third London edition. Philadelphia: Lindsay & Blakiston. 1874.

*Inflammation of the Lungs, Tuberculosis and Consumption. Twelve Lectures.* By LUDWIG BUHL, Professor of Pathological Anatomy and General Pathology in the University of Munich, etc., etc. Translated by permission from the Second German edition by Matthew D. Mann, M.D., and Samuel B. St. John, M.D. New York: G. P. Putnam's Sons. 1874.

*The Science of Homœopathy ; or, a Critical and Synthetical Exposition of the Doctrines of the Homœopathic School.* By CHAS. J. HEMPEL, M.D. Boericke & Tafel : New York, Philadelphia, Baltimore and San Francisco.

*The Breath, and the Diseases which Give it a Fetid Odor.* By JOSEPH W. HOWE, M.D., author of "Emergencies," Clinical Professor in the Medical Department of the University of New York, etc., etc. New York : D. Appleton & Co. 1874.

*On Cerebria and other Diseases of the Brain.* By CHAS. ELAM, M.D., London, Fellow of the Royal College of Physicians, etc., etc. Philadelphia : Lindsay & Blakiston. 1872.

PAMPHLETS RECEIVED.

*Transactions of Medical Society of New Jersey.* 1874.

*Report of* 105 *Cases of Operations for Cataract.* By B. JOY JEFFRIES, A.M., M.D., Ophthalmic Surgeon to the Massachusetts Charitable Eye and Ear Infirmary, etc.

*The Temperance Question from the Standpoint of the Present.* By JNO. MORGAN MCKOWN, M.D., Arcola, Ill.

*The Legal Relations of Emotional Insanity.* By E. LLOYD HOWARD, M.D., of Baltimore, Md. Extracted from the transactions of the American Medical Association.

CLINICAL CONTRIBUTIONS.

Three cases of Induration of the Os and Cervix Uteri, the result of Syphilis.

Two cases of Syphilitic Insanity.

Four cases of Anomalous Localities of Chancres—extra genital—with Remarks. By M. H. HENRY, M.D., Surgeon in Chief of the State Emigrant Hospital, Ward's Island, New York, etc., etc. 1874.

JOURNALS RECEIVED.

The American Medical Weekly—Oct. 24, 31, Nov. 7, 14.
" Atlanta Medical and Surgical Journal—November.
" American Journal of Insanity—October.
" American Journal of Syphilography and Dermatology—October.
" Boston Journal of Chemistry—November.
" Boston Medical and Surgical Journal—November.
" Cincinnati Lancet and Observer—November.
" Chicago Journal of Nervous and Mental Disease—October.
" Clinic, Cincinnati—Oct. 24, Nov. 14.
" Detroit Review of Medicine and Pharmacy—November.
" Druggists' Circular—November
" Eclectic Medical Journal—November.
" Indiana Journal of Medicine—November.
" Kansas City Medical Journal—November.
" Laboratory, Boston—October.
" London Lancet—October.
" Medical and Surgical Reporter, Philadelphia—Oct. 24, 31, Nov. 7, 14.

The Medical Times, Philadelphia—Oct. 17, 24, 31, Nov. 7.
" Medical Record, New York—Oct. 15, Nov. 2.
" Medical News and Library—November, and Supplement.
" Medical Press and Circular—October.
" Medical Examiner—Nov. 1.
" Nashville Journal of Medicine and Surgery—November.
" New Remedies, New York—October.
" New York Medical Journal—November.
" Pacific Medical and Surgical Journal—November.
" Peninsular Journal of Medicine—November.
" Practitioner, London—October.
" Pharmacist—November.
" Richmond and Louisville Medical and Surgical Journal—October.
" St. Louis Medical and Surgical Journal—November.
" Southern Medical Record—October.
" Sanitary Journal of Public Health—September.
" Virginia Medical Monthly—November.

